# Heat Shock Proteins in Alzheimer’s Disease: Role and Targeting

**DOI:** 10.3390/ijms19092603

**Published:** 2018-09-01

**Authors:** Claudia Campanella, Andrea Pace, Celeste Caruso Bavisotto, Paola Marzullo, Antonella Marino Gammazza, Silvestre Buscemi, Antonio Palumbo Piccionello

**Affiliations:** 1 Dipartimento di Biomedicina Sperimentale e Neuroscienze Cliniche (BIONEC), Università degli Studi di Palermo, Via del Vespro 129, 90127 Palermo, Italy; celestebavisotto@gmail.com (C.C.B.); antonella.marinogammazza@unipa.it (A.M.G.); 2 Dipartimento di Scienze e Tecnologie Biologiche, Università degli Studi di Palermo, Chimiche e Farmaceutiche (STEBICEF), Viale delle scienze Ed.17, 90128 Palermo, Italy; andrea.pace@unipa.it (A.P.); paola.marzullo@unipa.it (P.M.); silvestre.buscemi@unipa.it (S.B.)

**Keywords:** heat shock proteins, chaperones, Alzheimer’s disease, amyloid peptide, protein Tau, Hsp60, Hsp70, Hsp90

## Abstract

Among diseases whose cure is still far from being discovered, Alzheimer’s disease (AD) has been recognized as a crucial medical and social problem. A major issue in AD research is represented by the complexity of involved biochemical pathways, including the nature of protein misfolding, which results in the production of toxic species. Considering the involvement of (mis)folding processes in AD aetiology, targeting molecular chaperones represents a promising therapeutic perspective. This review analyses the connection between AD and molecular chaperones, with particular attention toward the most important heat shock proteins (HSPs) as representative components of the human chaperome: Hsp60, Hsp70 and Hsp90. The role of these proteins in AD is highlighted from a biological point of view. Pharmacological targeting of such HSPs with inhibitors or regulators is also discussed.

## 1. Introduction

Among neurodegenerative diseases, Alzheimer’s (AD) represents a major concern for public health in the 21st century. AD is mainly characterized by the anomalous processing of two proteins, amyloid-peptides (Aβ) and Tau, leading to the pathological formation of extracellular senile plaques and intracellular neurofibrillary tangles (NFTs). Brains of AD patients present senile plaques formed by insoluble Aβ with a sequence between 38 and 42 amino acids [1]. According to the *amyloid hypothesis*, Aβ peptides arise from the β-amyloid precursor protein (APP). APP is a trans-membrane protein which is cleaved by β- and γ-secretase. The former—also known as beta-site amyloid precursor protein cleaving enzyme (BACE-1)—produces sAPP β, which is a soluble amyloid precursor and a C-terminal fragment (C99) bound to the membrane [1]. In turn, the cleavage of C99 by γ-secretase releases Aβ_40_ and Aβ_42_. Both peptides tend to self-assemble into oligomers and then into fibrils. In particular, Aβ_42_ is the major component in amyloid plaques and forms the most toxic oligomers. As a consequence, an increased production of Aβ induces cell death, eventually leading to dementia [2]. On the other hand, the intra-cellular NFT lesion results from the pathological hyperphosphorylation of protein Tau and its subsequent misfolding, aggregation and accumulation within the cytoplasm [3].

Currently, the only approved therapy is focused on the limitation of symptoms by inhibiting acetylcholinesterase (AChE) action, thus enhancing cholinergic transmission [4]. Due to the involvement of APP metabolism in Aβ production, the inhibition of secretase enzymes represents a very promising strategy for AD treatment and clinical candidates are in phase 3 trials [5]. On the other hand, many other therapeutic approaches are under evaluation. In particular, Aβ peptide and Tau aggregation inhibitors, photo-therapeutics and metal chelators are among the most promising lead but, currently, these approaches are far from being implemented in clinical practice [6,7,8,9,10,11]. 

Considering that the main cause of neuron’s damage in AD is due to stress induced by the misfolding of Aβ peptides and Tau, triggering the production of toxic oligomers and eventually plaques and NFTs, the importance of the chaperones in AD and other neurodegenerative diseases has been evidenced in the last two decades. [12]. Among molecular chaperones, Heat Shock Proteins (HSPs) are major constituent of the chaperome and Hsp60 [13], Hsp70 [14,15,16] and Hsp90 [17,18] are considered target [19] of particular relevance in AD [20] and for many other diseases, including cancer [21,22,23,24]. In this review, recent advances regarding the role of these three proteins in AD is highlighted from a biological point of view. The state of the art of their targeting and the development of perspective drugs for future AD therapies is also discussed.

## 2. Molecular Chaperones and Neurodegenerative Diseases

To face stress, cells use a series of protective mechanisms. One of these biological responses to stress involves an array of highly conserved proteins that have a range of functions with the scope of maintaining cellular homeostasis [25]. These biomolecules include a group of protein named molecular chaperones that play a crucial role within cells, by mediating protein folding, signalling, chaperoning and cell protection. These proteins, that are located inside cells as well as in extracellular environment and in body fluids, are important players in other cellular mechanisms such us protein translocation, protein degradation, cell differentiation and signal transduction [26,27,28]. The expression of many chaperone proteins is induced by stress to assist other proteins in achieving proper folding. These molecular chaperones are included in the family of HSPs however, not all molecular chaperones are stress proteins [29]. There are several classes of HSPs involved in the system to assure the control of protein quality: Hsp60, Hsp70, Hsp90, Hsp40, Hsp100 and Hsp110 as well as the ATP independent small HSPs such as Hsp20 [30]. In this context, chaperonopathies are pathological conditions in which chaperones that are abnormal in composition/structure (e.g., because of mutations or post-translational modifications), quantitative levels, location, or function, play an either primary or auxiliary etiopathogenic role [21]. 

The accumulation of misfolded proteins and protein aggregation in the human brain is an important characteristic of many neurodegenerative diseases, including AD, Amyotrophic lateral sclerosis, Parkinson’s disease (PD), Huntington’s disease and Creutzfeldt-Jakob disease [31,32] (Table 1). Therefore, neurodegenerative disorders are classified among “proteinopathies,” in which proteins that are misfolded (i.e., conformationally altered) can direct disease progression and are often used as a primary neuropathological biomarker of the disease. 

Neuronal dysfunction caused by the abnormal aggregation of proteins is a crucial factor for the medical evaluation of these neuronal diseases. The clinical characteristics depend on the affected brain region and may involve disruption of daily activities including sensory, motor and cognitive functions. One hypothesis is that misfolding and protein aggregation cause synaptic loss and neuronal death which are typically observed in various neurodegenerative diseases [31]. The aggregation of misfolded proteins is highly regulated and depends on genetic and environmental factors [31]. 

**Figure 1:**
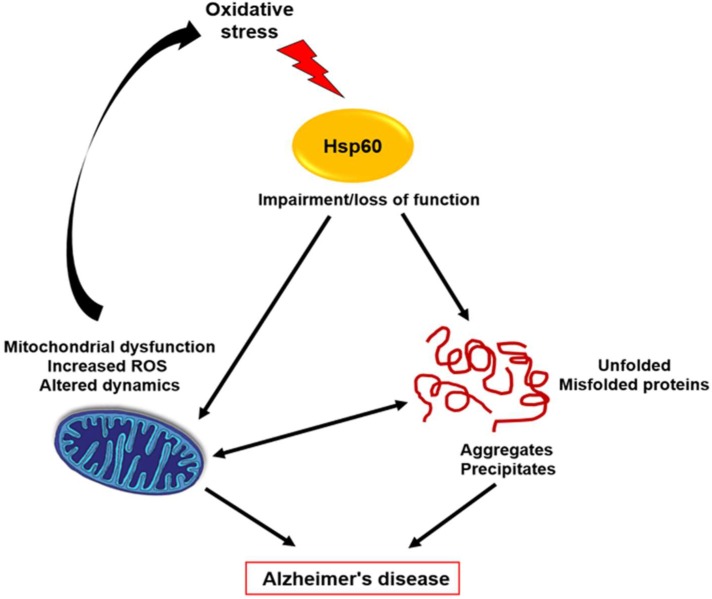
Hsp60 and Alzheimer’s disease. Oxidative stress may cause Hsp60 structure modifications leading to loss of Hsp60 functions with the consequences of protein misfolding, aggregation and deposition.

Molecular chaperones regulate protein folding, modulate protein activity and target misfolded or aggregated proteins for refolding or for degradation and translocation. HSPs are essential to efficiently facilitate the protein folding process [36]. They participate in different mechanisms to defend the cells against stress-related events harmful to the cell itself [36]. Therefore, as observed in various neurodegenerative diseases, failure of these cellular control mechanisms can result in pathogenic conditions. There are many data that demonstrated that HSPs regulate protein misfolding in a variety of neurodegenerative diseases, such as AD, probably displaying protective roles and/or acting as pathogenic factors. Indeed, stress-induced proteins like chaperones have been claimed to act as protective molecules for cells of the nervous system [20]. Many evidences demonstrated that oxidative stress is a feature of AD and PD [37]. Mitochondrial dysfunction and abnormal accumulation of Aβ and Tau proteins can contribute to create the imbalance between oxidant and antioxidant mechanisms determining oxidative damage in AD patients [37]. In the brain, oxidative stress can cause damage that contributes to neuronal loss [38]. Reactive oxygen species (ROS) can accumulate inside cells and have negative effects on all biological molecules, determining, for instance, nucleic acid breakage, enzyme inactivation, polysaccharide depolymerisation and lipid peroxidation. Under these stress conditions, the expression of the genes encoding HSPs was induced [38]. Moreover, mitochondrial dysfunction and elevated levels of ROS might create a vicious circle contributing to AD instauration and progression (see Figure 1) [20]. 

Indeed, various HSPs can be transported to synapses and axons to block or hinder the aggregation process of misfolded proteins. Many data demonstrated that HSPs have a role in the direct inhibition of the aggregation of amyloidogenic proteins and also promote bonding to ubiquitin and degradation of aggregated or misfolded proteins [39,40]. Many evidences implicate HSPs in metabolism and the aggregation of both Aβ and Tau [41]. HSPs that are found in the mitochondria matrix can play an important role in protein folding. Alterations in HSPs function affect mitochondria function, such as protein aggregation [42]. It is known that intracellular protein degradation pathways are decreased with aging in many tissue and organs. Indeed, in several neurodegenerative diseases, the protein degradation system is not functional. Moreover, HSPs are involved in a specialized mechanism called chaperone mediated autophagy (CMA). CMA is the only autophagic pathway that allows selective degradation of soluble proteins that contain a consensus peptide motif in lysososmes [43,44].

## 3. Hsp60

### 3.1. Biological Role in AD

Hsp60 is a protein that, together with its co-chaperone Hsp10, is considered essential for mitochondrial protein folding [36]. Many studies have demonstrated that Hsp60 can be localized in extra-mitochondrial sites such as in the cytosol, in extracellular vesicles, or on the surface of normal and tumour cells [45,46,47]. Recently, increasing data demonstrated that Hsp60 is localized outside of the cells, where it mediates the interaction between immune cells and other body tissues [48]. Hsp60 can have both pro-survival and pro-death functions depending on the molecules with which it interacts, on the tissue, on the cell type and on the identity of the apoptosis inducers. [27,49,50,51,52,53]. Many evidences have demonstrated that Hsp60 have a role in tumour progression as suggested by its accumulation in the cytosol and plasma membrane of cancerous cells [21]. We also demonstrated that Hsp60 can be secreted in the extracellular space via secretory vesicles that, in turn, can modulate anti-tumour immune responses [45,46,47]. Many researchers have advanced the hypothesis that Hsp60 can be used as a target for anticancer therapy and data in the literature are very encouraging in this regard [21]. For instance, hyperacetylation of Hsp60 in osteosarcoma cells is associated with the anticancer activity of geldanamycin, and Hsp60 nitration is associated with the anti-tumour action of the histone deacetylase inhibitor SAHA in mucoepidermoid cells [26,54]. On the other hand, Hsp60’s role in AD is still unclear. Many data demonstrated that it has a neuroprotective role but other authors have attributed a deleterious effect to the elevated expression of Hsp60 in AD [55,56]. It has been demonstrated that Hsp60 expression by activated microglia is high. Moreover, the extracellular release of Hsp60 increases the production of other pro-inflammatory factors through binding to toll-like receptor 4 (TLR-4) and stimulating neuronal cell death [55]. Over-activation of microglia in response to certain harmful factors, contributes to the progression of several neurodegenerative diseases, including AD [57]. Neurodegenerative diseases are associated with the secretion of various pro-inflammatory and cytotoxic factors by activated microglia in the brain [58,59]. Therefore, inhibiting the activation of microglia and Hsp60 expression/release is an important strategy for the prevention of neurodegeneration [57]. Hsp60 levels were high in lymphocytes from AD patients when compared to controls [60,61]. Indeed, a useful approach would be the test of Hsp60 levels in patients with clinical condition preceding AD, such as mild cognitive impairment, in order to assess the potential value of this protein as an early biomarker of the disease [60,62]. Mitochondrial protein quality control may have a special relevance for the maintenance of neurons. Mitochondrial dysfunction was found in numerous neurodegenerative diseases including AD, HT and Parkinson’s disease [63]. Mutations in mitochondrial genes or nuclear genes encoding mitochondrial proteins are potential causing of neurological diseases and defects of the mitochondrial protein quality control system could represent an important pathogenic factor for neurodegenerative diseases. In particular, mutations in the gene encoding for Hsp60 are associated with hereditary spastic paraplegia (SPG13) [63]. Proteomic analysis of hippocampi of APP-transgenic mice possessed abundant Aβ oligomers from the age of 8 months but no amyloid plaques even at the age of 24 months and showed altered levels of 14 proteins including Hsp70, Hsp60 and Hsp90. In particular, Hsp60 and Hsp70 levels were significantly decreased with respect to control. Aβ oligomers might contribute in changing the expression of the chaperons [56]. Aβ, is the main component of plaques and it accumulates in mitochondria collected from brains of human AD cases and transgenic mouse models of AD [64]. It was demonstrated that HSPs played a protective role in cultured neurons. In particular, Hsp60, Hsp70 and Hsp90 either individually or together, provide protection against intracellular beta-amyloid induced stress through the maintenance of mitochondrial oxidative phosphorylation and functionality of tricarboxylic acid cycle enzymes. Aβ selectively inhibits complex IV activity and such inhibition is selectively neutralized by Hsp60. In this way, the overall effect of HSPs activity resulted in the reduction of free radicals, preservation of ATP generation, reduction of cytochrome C release and prevention of caspase-9 activation, all involved in beta-amyloid-induced neuronal dysfunction and death [65]. On the contrary, it was shown that Hsp60 mediates in vitro the translocation of APP to the mitochondria, leading to dysfunction of this organelle. In particular, Walls et al. found that Hsp60 and APP/Aβ form a molecular association in mitochondria in both transgenic and human AD subjects [41]. Immunoprecipitated APP from human AD mitochondria exhibited a stronger propensity to interact with Hsp60 versus non-demented controls. Mangione et al. [66] demonstrated, in vitro, that Hsp60 inhibits Aβ amyloid aggregation by closing molecular pathways leading to peptide fibrillogenesis. Administration of an Aβ amyloid-Hsp60 peptide-conjugate vaccine led to the induction of anti-Aβ-specific antibodies, associated with a significant reduction of cerebral amyloid accumulation in a mouse model of AD [67]. All these experimental evidences made us hypothesize that the regulation of Hsp60 production could have been a potential therapeutic option for the treatment of AD. However, Hsp60 role in AD remains controversial and further investigations are necessary to better understand if this protein is either a “friend” or a “foe” in the development and progression of the disease [20].

### 3.2. Targeting and Inhibition

Despite the convincing evidence that supports the involvement of Hsp60 in the development of Alzheimer’s disease [41], there is a lack of studies on its known inhibitors or regulators that could represent potential therapeutic agents in AD. On the other hand, Hsp60’s role in tumours has been assessed and studies regarding the development of inhibitors are related to the opportunity of targeting Hsp60 as a therapeutic anticancer approach [21,22,68]. These compounds are able to modify and regulate Hsp60 expression and functions and, for this reason, their use can be switched from cancer therapy to AD management [20]. In general, various studies pointed out Hsp60 inhibition as a promising therapeutic approach but only a limited number of compounds have been fully characterized and, for most of these inhibitors, the mechanism of action is still undisclosed [69].

In the search of new Hsp60 inhibitors, it is fundamental to consider structural differences between the eukaryotic Hsp60 and its prokaryotic homologue GroEL. Only the first possesses cysteine residues (Cys237, Cys442 and Cys447), which represent ideal drug-interacting sites due to their nucleophilic behaviour and redox potential [21]. Moreover, from a structural point of view, the X-ray structure of Hsp60 was only recently resolved [70] and the models for the Hsp60 folding machine are still under debate considering: the bullet versus football complex with Hsp10 co-chaperones [71,72]; one-ring heptamers versus two-ring tetradecamers [73,74]; the significant differences between crystal and in solution structures [74]. The overall complexity of these aspects accounts for the lack of a model that could be used for drug design and in silico screening, thus slowing down the drug discovery process. Currently, only two modes of action were described for Hsp60 inhibitors: competition with ATP binding site or targeting cysteine residues [21]. 

**Figure 2:**
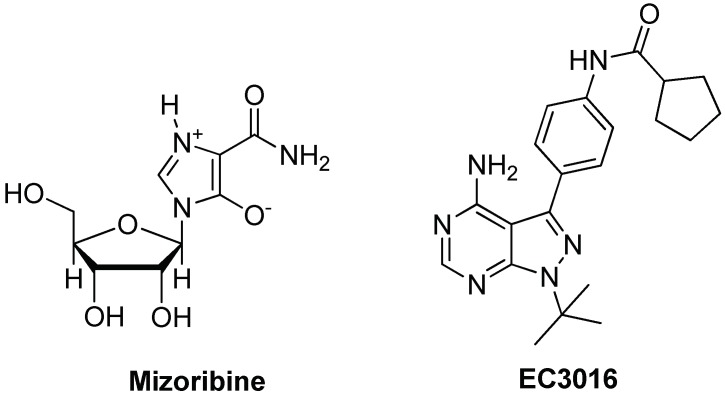
Chemical structures of Hsp60 inhibitors binding the ATP binding site.

The first example of a compound targeting the ATPase activity was mizoribine, an imidazole-based immunosuppressant (see Figure 2), able to complex with Hsp60, thus affecting its protein-folding activity [75,76]. Interestingly, mizoribine’s activity was also related to the inhibition of the detachment of the co-chaperonin Hsp10 from the Hsp60/Hsp10 complex with a significant difference in the activities observed with the prokaryotic GroEL/GroES system, which is not significantly affected by mizoribine [77]. The pyrazolopyrimidine EC3016 (see Figure 2) was also reported to inhibit the protein-folding function of Hsp60 by blocking ATP binding and hydrolysis [76].

**Figure 3:**
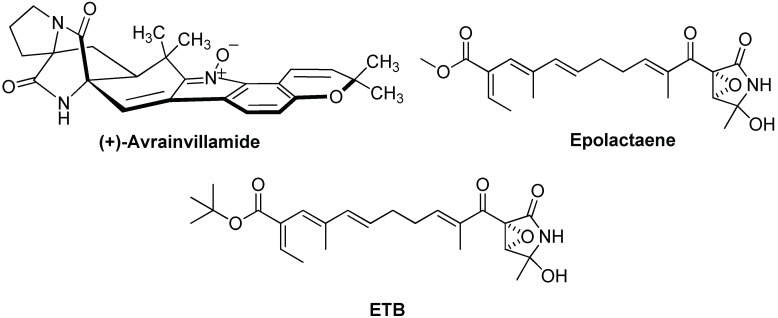
Chemical structures of Hsp60 inhibitors binding the Cys442 residues.

Among compounds that are supposed to interact with cysteine residues, it is worth mentioning natural compounds such as avrainvillamide and epolactaene (see Figure 3). Avrainvillamide is a fungal metabolite supposed to alkylate Hsp60’s cysteine residues through the electrophilic indole-oxide moiety [78]. However, its mode of action has not been demonstrated yet.

One of the most studied Hsp60 inhibitors is epolactaene, a bacterial metabolite which inhibits the folding activity of human Hsp60, through covalent binding to Cys442 [79]. Epolactaene derivatives, such as the tertiary butyl ester **ETB** (Figure 3), were also active [80] and SAR studies demonstrated that both the cyclic amide (lactam) and the α,β-unsaturated ketone are critical moieties for inhibiting the chaperone activity of Hsp60 [81]. An in-silico study suggested a putative mode of action for epolactaene, revealing the opening of a binding pocket in proximity of Cys442 after ATP occupies its binding site and that epolactaene covalently binds thiol moiety of Cys 442 through attack at C14 and epoxide ring-opening [82]. MD studies evidenced that epolactaene binding hinders the dynamic conformational changes of the monomer necessary for functional folding process.

**Figure 4:**
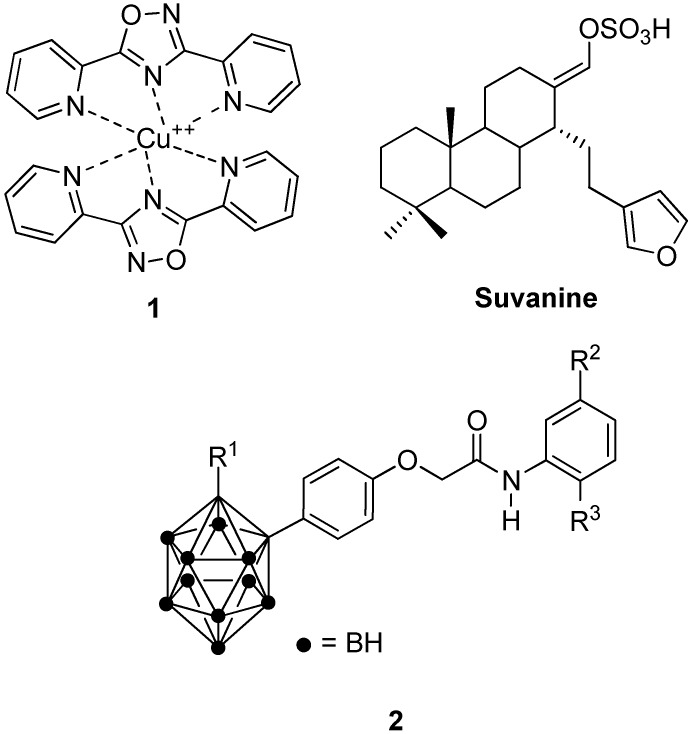
Chemical structures of Hsp60 modulators.

Other representative molecules interacting with Hsp60 or affecting its expression with unknown mechanism of action include copper Complex **1** [27,83], marine sesquiterpene suvanine [84] and carboranylphenoxyacetanilide derivatives **2** [85,86,87] (Figure 4). Other portions of Hsp60 can be investigated to develop new inhibitors; for instance, targeting could be focused on the site of interaction between the mitochondrial Hsp60 and Hsp10. Overall, lack of consensus on the oligomers involved on the folding cycle and the lack of a co-crystallized structure with a known inhibitor, leave several unanswered questions concerning Hsp60’s role in AD. Drug design targeting Hsp60 is therefore a perspective growing field of research and its translation into potential AD therapies is still unexplored.

## 4. Hsp70

### 4.1. Biological Role in AD

The Hsp70 family is composed by 17 members [32], some of which can be induced by stress while others, such as Hsc70, are constitutively expressed. Hsp70 chaperones are found in most cellular compartments, including the nucleus and cytoplasm (Hsc70), mitochondria (mtHsp70, also known as HSPA9 or mortalin) and ER (Grp78, also known as BiP) [32,88]. Hsc70 assists the folding of client proteins via an ATP dependent mechanism and prevents aggregation of the unfolded proteins [88]. Hsp70 can be associated with the co-chaperones Hsp40 and can also collaborate with Hsp90 in various cellular compartments [89]. Overexpression of Hsp70 can determine resistance against apoptosis-inducing agents while downregulation of Hsp70 levels leads to increased sensitivity towards these agents [90,91]. Hsp70 levels were increased in different type of tumours and its presence is associated with poor prognosis in breast and endometrial cancer. Hsp70 binds tumour-suppressor proteins, determining unlimited cellular growth and increased resistance to chemotherapy in breast cancer [88,92]. On the contrary, downregulation of Hsp70 levels in some types of cancers induces differentiation and cell death [93]. Hsp70 can trigger the activation of the immune response by stimulating both innate and adaptive immunities. Moreover, Hsp70 is actively secreted by different types of cells via unusual protein secretory routes, including exosome pathways [94]. Extracellular Hsp70 can exert an immunomodulatory effect, thus playing an important role in the immune response to cancer cells [95,96]. For instance, microvesicles bearing Hsp70 on their surface can activate macrophages or other natural killer cells and play as an indirect regulator of vascular homeostasis [94,97]. 

Many data demonstrated that Hsp70 is involved in neurodegeneration. In the brains of transgenic mice affected by AD, an increased level in the expression of Hsp70 has been associated with protective effects [98]. Hsp70 may accomplish a neuroprotective role, inhibiting Aβ aggregation suggesting a potential role of Hsp70 in the pathogenesis of this disease [99]. Indeed, Hsp70 can bind with APP and interfere with its secretory route to reduce formation of Aβ [31]. Additionally, Hsp70 can degrade Tau and Aβ oligomers trough the proteasome system [31]. Immunohistochemistry assays and protein expression analyses in AD brain tissues showed high levels of Hsp70 expression in affected regions and these levels appeared to be correlated to the presence of activated glia and dysregulated or stressed neurons [100]. The combination of Hsp70/Hsp40 and Hsp90 induces structural modifications in cytosolic Aβ oligomers but has little effects on fibrils [43]. There are two proposed mechanisms by which HSPs can inhibit the aggregation of Aβ. In one pattern, the chaperone binds misfolded amyloid in an ATP-independent manner, preventing it from aggregation. In a second pattern, the chaperone may bind Aβ in an ATP-dependent manner, changing Aβ conformation to one that is less susceptible to aggregation [101]. Cumulative evidence indicates that Hsp70 has neuroprotection activity against various intracellular amyloids in Drosophila and mouse models [102]. Hsp70 has been associated also with extracellular deposits in AD. In fact, while Hsp70 is normally a cytosolic protein, such an association may be a consequence of release, probably through exosomes to stop the accumulation of proteotoxic assemblies, in agreement with the increased levels of Hsp70 observed in AD. De Mena et al. [102] demonstrated that the engineered form of secreted Hsp70 is highly protective against toxicity induced by extracellular deposition of the Aβ_42_ in Drosophila. Chaperone proteins, including Hsp70, can bind abnormal Tau directly and reduce its concentration by favouring its degradation and de-phosphorylation [32]. Overall, it is clear that the Hsp70 family is implicated in AD through pathogenic and/or protective mechanisms in which these chaperones (with or without their co-chaperones) participate.

### 4.2. Targeting and Inhibition

**Figure 5:**
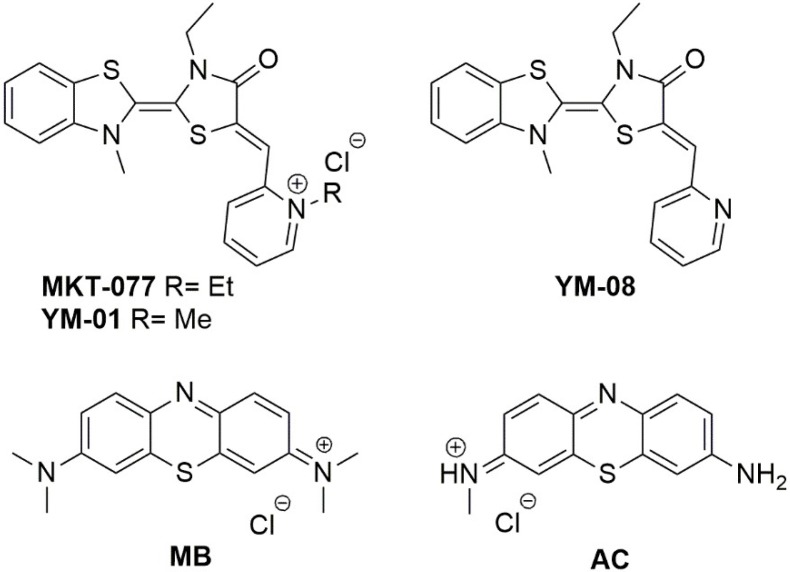
Chemical structures of Hsp70 inhibitors targeting the ATP binding site.

The drug discovery studies targeting Hsp70’s role in AD mainly consists of the transpositions of previous researches in cancer therapy. Moreover, researchers could benefit of well-established screening strategies of new compounds in vitro, as well as in vivo [103]. Hsp70 targeting is related to the design of inhibitors pointing at the ATP-binding site, that is, the allosteric sites in the nucleotide-binding domain (NBD), which is also the substrate-binding domain (SBD) [19]. Compounds bearing the (benzothiazolin-2-yliden)-4-oxothiazolidin-2-ylidene (rhodacyanine) skeleton are reported to bind different allosteric sites of Hsp70 and were previously investigated as anti-cancer compounds [19]. Among these, homologues MKT-077 and YM-01 (Figure 5) were considered as candidate in AD treatment for their ability to rapidly and potently reduce Tau levels in vitro and ex vivo, by targeting Hsp70 [104,105].

In Tau transgenic brain slices, YM-01 also increased long-term potentiation. Even if its mode of action and binding site was extensively studied by means of NMR and computational techniques [104], MKT-077 was not further considered due to its renal toxicity and low BBB penetration, in general, the rhodacyanine scaffold was considered difficult to improve. Nevertheless, this scaffold was considered as one of the most promising for the development of multimodal drugs able to reduce Tau levels through Hsp70’s modulation to interact with misfolded Tau, thus reducing its toxicity [106]. Indeed, one major improvement of this scaffold was the removal of the pyridinium moiety, as in inhibitor YM-08 (see Figure 5), bearing a neutral pyridine ring [107]. Compared to MKT-077, YM-08 showed a lower activity concerning Hsp70 inhibition and Tau/phospho-Tau reduction but possessed a better PK profile, due the ability to cross the BBB [107].

**Figure 6:**
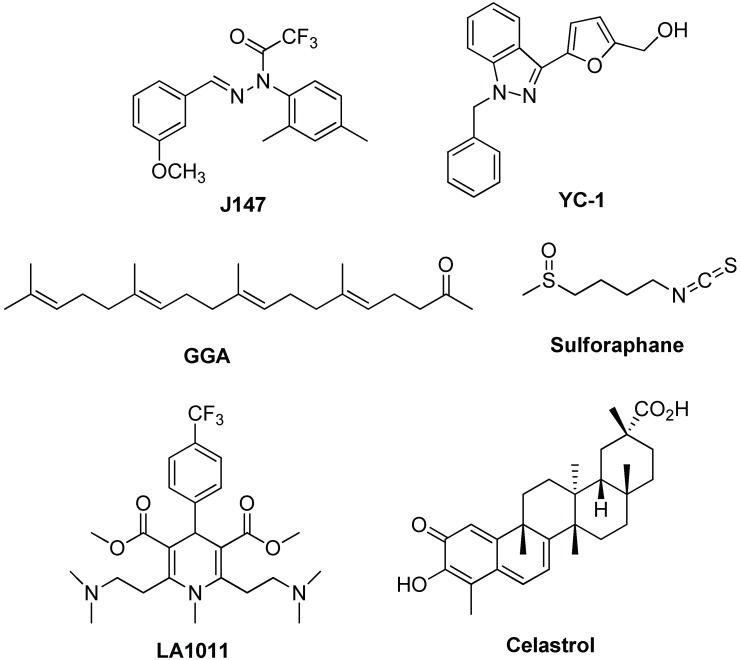
Chemical structures of modulators of Hsp70 expression.

Another important class of molecules is that of phenothiazines (see Figure 5). For instance, Methylene Blue (MB) and Azure C (AC) are able to reduce total Tau and phosphorylated-Tau levels through the inhibition of Hsp70 ATPase function, although with low selectivity [108,109]. Tau toxicity reduction was observed when AC directly interacted with toxic oligomers, by means of induced conformational changes [110], an effect observed also for MB [106]. This suggests the use of phenothiazine derivatives as multimodal drug toward AD. Moreover, the synergistic effect of Hsp70 ATPase activity and Tau aggregation inhibition seems a good way for therapeutic intervention and these two targets should be combined during drug screening [106]. Other representative molecules able to modulate Hsp70 expression are reported in Figure 6. 

**J147** is a potent neurotrophic molecule that, in a transgenic AD mouse model, prevents the loss of synaptic proteins and cognitive decline by reducing Hsp70 expression, while inducing Hsp90 overexpression [111]. YC-1, a synthetic small molecule initially developed as an activator of guanylyl cyclase (GC), was proposed as neuroprotective compound due to its ability to suppress Aβ_25–35_ toxicity in PC12 cells by inducing Hsp70 overexpression [112]. Moreover, geranylgeranylacetone (GGA), a drug approved for ulcer therapy, is able to induce Hsp70 expression with a safe profile. It was tested in an APP23 AD mice model improving its cognitive function and decreasing levels of Aβ, Aβ plaque deposition and synaptic loss [113]. Initially, GGA mode of action was unclear and in some experiments demonstrated to be HSP-independent. More recently, it was verified that amelioration in AD model occurs by regulation of the ERK/p38 MAPK signalling pathway [98]. Oral treatment of a triple transgenic mouse model of AD (3 × Tg-AD) with sulforaphane increases levels of Hsp70 and C-terminus of Hsp70-interacting protein (CHIP), inducing Aβ and Tau clearance and restoring memory deficits [114]. Similar positive effects were also evidenced for 1,4-dihydropyridine candidate LA1011, a synthetic molecule able to upregulate Hsp70 in vitro in SH-SY5Y cells and in vivo with a APPxPS1 mouse model of AD [115]. The extract of Ginkgo biloba leaves, an accepted traditional Chinese medicine, reduce neurotoxicity of the Aβ_1–42_ oligomer by increasing Hsp70, among other proteins, in SH-SY5Y cells [116]. Similarly, the Hsp70-induced effect was demonstrated in a neuronal cellular model for celastrol [117]. In general, targeting of Hsp70 seems a good strategy in the search of neuroprotective drugs, in particular, for the managing of Tau in AD and in other tauopathies. Further advances in this field could be envisaged in the next future with selective targeting of constitutive protein *versus* stress-induced ones.

## 5. Hsp90

### 5.1. Biological Role in AD

**Figure 7:**
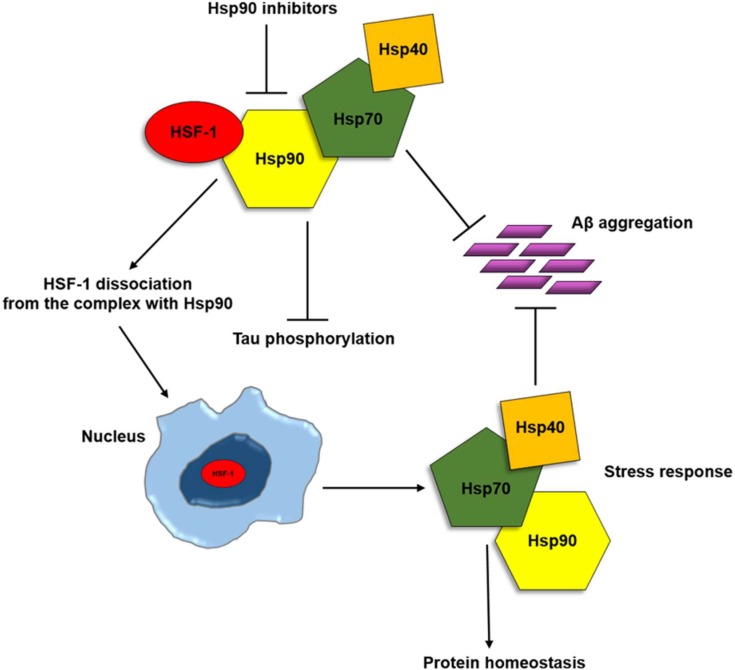
Hsp90 inhibition in Alzheimer’s disease. Hsp90 down regulation may induce the reduction of Tau hyperphosphorilation and aggregation and may trigger the so-called stress response. In fact, in the presence of cellular stress and Hsp90 inhibitors, Heat Shock Factor 1 (HSF-1) dissociates from the chaperone, reaches the nucleus, inducing the activation of heat shock genes and of the stress response via the production of Hsp90, Hsp70 and Hsp40, restoring protein homeostasis.

There are at least five types of human Hsp90: HSP90A in cytosol, HSP90alpha, HSP90beta, HSP90B (or Grp94) in the ER and TRAP in mitochondria [118]. Under stress conditions, Hsp90 is the most abundant protein in eukaryotic cells and, like other molecular chaperones, is present in any of its form in most cellular compartments (cytosol, endoplasmic reticulum, mitochondria and chloroplast) [118]. Hsp90 is an ATP-dependent chaperone and plays an important role in the folding of many proteins and in the refolding of denatured proteins after stress [32]. Hsp90 binds several substrates in their native states and targets a specific set of client proteins that are involved in signal transduction [113]. Many of these client proteins are bound to Hsp90 in an inactive state and are activated upon dissociation from Hsp90 [118]. Hsp90 interacts with important client kinases, including cyclin-dependent serine kinases [118]. In cancer cells, Hsp90 is overexpressed and is essential for the malignant transformation and progression of several tumour types such as bladder, breast and lung cancers, as well as leukaemia [30]. Similar to Hsp60 and Hsp70, also Hsp90 has a role in AD. Many data demonstrated that Hsp90 inhibits amyloid aggregation [43], while the complex of Hsp90 with Hsp70/Hsp40 can inhibit Aβ formation [43]. Hsp90 can be released in extracellular environment free or associated with exosomes [94]. When outside the cell, it has a role in activating the immune system [97]. In nervous system, extracellular Hsp90 determines activation of microglial phagocytosis that push Aβ degradation by activation of the Toll-like receptor-4 (TLR4) pathway [119]. From another point of view, chaperone proteins such as Hsp90 form macromolecular complexes with co-chaperones, which can regulate Tau metabolism and Aβ processing [32]. Many data demonstrated that pharmacological inhibition of Hsp90 significantly decreases intracellular levels of the disease-associated phosphorylated Tau species via proteasomal degradation [100]. Administration of Hsp90 inhibitors to primary neurons prevented Aβ induced neurotoxicity [120]. Dickey et al. [121], demonstrated that inhibition of Hsp90 determined a reduction of phosphorylated Tau form and the carboxy terminus of Hsp70-interacting protein (CHIP) is involved in this mechanism. The recruitment of CHIP protein, a co-chaperone with E3 activity, induces the ubiquitination of Tau protein and activates its downstream degradation processes. Many data demonstrated that the combination of chaperones was able to significantly affect the aggregation (see Figure 7).

### 5.2. Targeting and Inhibition

**Figure 8:**
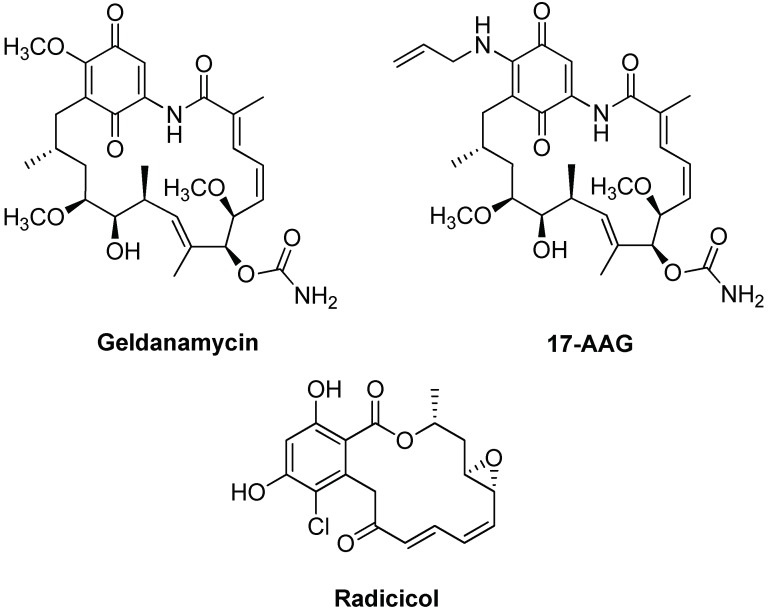
Chemical structures of Hsp90 inhibitors targeting the ATP binding site.

Contrary to Hsp60 and Hsp70, Hsp90 role in AD development and progression seems better defined as reported in the literature cited above. For example, Hsp90 inhibition might be useful in AD treatment counteracting Tau protein hyperphosphorylation and aggregation. However, also in this case, the research of Hsp90 inhibitors in AD could benefit from previous findings regarding anti-cancer drugs [122], with many compounds already tested in clinical trials [123]. The identification of potential Hsp90 inhibitors could be efficiently performed by means of different screening methods including microarray- [124], virtual- [125,126] or cell-based screening [127]. Hsp90 inhibitors mainly interact with the nucleotide-binding pocket, located in the N-terminal domain, where they bind to the ATP-binding site preventing the ADP- and ATP-bound conformational changes necessary for the chaperone activity [19]. This protein site is targeted by many interesting inhibitors, such as Geldanamycin (GA), 17-allylamino-17-desmethoxy-geldanamycin (17-AAG) and radicicol (see Figure 8). 

GA was the first discovered Hsp90 inhibitor; it was isolated from Streptomyces genus and was initially studied as antibiotic and antitumor but toxicity issues stopped further studies [123]. Nevertheless, many GA analogues were developed and 17-AAG was particularly considered as a potent Hsp90 inhibitor with better solubility and safer profile. Pharmacokinetic data obtained from research on 17-AAG as anti-tumoral drug, induced its repurposing as a therapy against AD and other neurodegenerative diseases. The in vivo effects of 17-AGG were demonstrated in a rat model, injected with Aβ_25–35_ into the hippocampus. [128]. Oral administration of 17-AAG reduces brain injury and improves cognitive processes by inducing HSPs (Hsp27, Hsp40 and, in particular, Hsp70) overexpression at the cellular level. The effect of this inhibitor on the other major target in AD, Tau, was tested in vivo in a mouse model, revealing that high dose of 17-AAG tended to decrease NFTs in transgenic mice [129]. Interestingly, these studies evidenced no effect of 17-AAG on amyloid plaques in Tg2576 mouse model and a significant reduction of NFTs in male tau transgenic (JNPL3) mice. Other authors demonstrated, in Tg2576 mouse model and cultured neurons, that 17-AAG reduces the damage from soluble Aβ and activates the expression of synaptic proteins through HSF1 [130]. In a model of Drosophila larvae expressing human Tau, the protein was reduced in larvae treated with 17-AAG but without the ability to restore locomotion deficit [131]. A similar trend was observed for radicicol, which was proposed for the treatment of neurodegenerative diseases [132]. 

**Figure 9:**
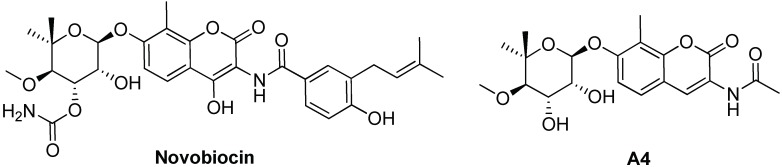
Chemical structures of Hsp90 inhibitors binding the C-terminal domain.

Hsp90 C-terminal domain was also described as an important target, even if few inhibitors are reported in the literature, such as celastrol (see Figure 6), novobiocin and its derivative A4 (see Figure 9) [19]. The protective effect of these compounds was demonstrated on cellular models through Aβ-induced cell death experiments [133,134]. For novobiocin-derived compound A4, the ability to modulate Hsp70 expression as well as a simulation of BBB penetration was also reported [134].

**Figure 10:**
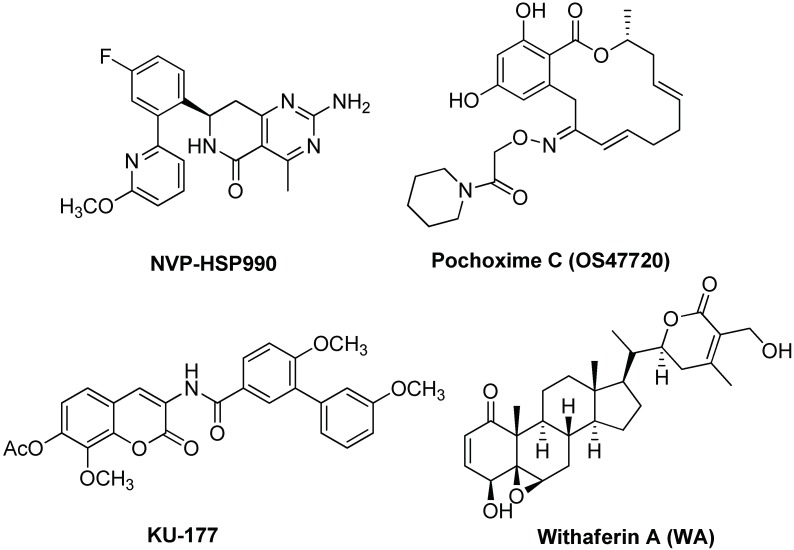
Chemical structures of Hsp90 inhibitors/modulators.

Other Hsp90 inhibitors or modulators were recently investigated (see Figure 10). Reversal of synaptic impairments in a rTg4510 transgenic AD mouse model was obtained with compound NVP-HSP990 which has a high Hsp70 induction capacity and is probably able to induce Tau clearance [135]. Pochoxime C (OS47720), a CNS-permeable and non-toxic Hsp90 inhibiting compound, restores synaptic dysfunction and memory loss in vivo in a Tg2576 mice AD model [136]. The effects of OS47720 depend upon HSF-1 activation and are followed by HSF1-mediated transcriptional events on synaptic genes. This study points out the importance of using Hsp90 inhibitors with a safe profile for an actual application toward neurodegenerative diseases and suggests their use in association with other drugs, such as β-secretase inhibitors, for a perspective multiple drug therapy approach. 

Finally, a recent trend is the modulation of Hsp90 functions through co-chaperones modulations. In fact, the co-chaperone activator of Hsp90 ATPase homolog 1 (Aha1) increased the production of aggregated Tau [137]. Treatment with KU-177, a novobiocin-based Aha1 inhibitor, reduced the accumulation of insoluble Tau in rTg4510 transgenic mouse model [137]. Similarly, a potential application for AD treatment was suggested for withaferin A (WA), a potent inhibitor of the Hsp90/Cdc37 interaction by regulation of LRRK2, like celastrol [138].

## 6. Conclusions

The study of connections between AD and HSPs is a research area of great interest and therapeutic potential in the next future (Table 2). 

In the last decade, many results were obtained mainly from research on anti-cancer agents. Some compounds of potential therapeutic interest were highlighted but clinical trials are not in due course. Therefore, gaining further knowledge is fundamental and many issues should be clarified, such as: (i) AD biochemical pathways involving HSPs; (ii) mode of action of HSPs inhibitors; (iii) selective targeting of constitutive versus stress-induced HSPs; (iv) understanding of client/HSPs protein-protein interactions at the molecular level [139,140]. In general, HSPs targeting could be a keystone for perspective drugs in the context of multitargeted drug discovery and polypharmacological approach toward a complex disease such as AD.

## Figures and Tables

**Table 1:** Neurodegenerative diseases due to protein misfolding and aggregation.

Diseases	Proteins Involved	Reference
AD	Aβ and Tau	[20]
PD	α-Synuclein and Tau	[3]
Huntington	Huntingtin	[33]
Prion	PrP	[34]
Taupathies	Tau	[3]
Lewy bodies dementia	α-Synuclein and ubiquitin	[35]

**Table 2:** HSPs localization, functions and involvement in AD and neurodegeneration.

HSPs	Localization	Association	Functions	CNS Diseases	Pharmacological targeting
Hsp60	- mitochondria; - extra-mitochondrial localization; - extracellular environment [45,46,47,94].	- APP/Aβ [41,66,67].	- mitochondrial protein folding [36]; - both pro-survival and pro-death functions depending on the molecules with it interacts [27,49,50,51,52,53]; - interaction between immune cells and other body tissues [48].	- hereditary spastic paraplegia (SPG13) [63]; - AD [64].	- competition with ATP binding site [21,76]; - targeting cysteine residues [21,78,79,80,81,82]; - inhibition of protein-folding activity [75,76]; - inhibition of the dissociation of the co-chaperonin Hsp10 [77]; - interaction with unknown mechanism [27,83,84,85,86,87].
Hsp70	- cytoplasm; - ER; - nucleus; - mitochondria; - extracellular environment [32,88,94].	- APP [31]; - tau [32].	- folding of client proteins; - prevention of aggregation of the unfolded proteins [88]; - immuno-modulatory effects [95,96].	- AD [31,98,99,100].	- binding of different allosteric sites of Hsp70 [19] through its modulation to interact with misfolded Tau [104,105,106]; - modulation of Hsp70 expression levels [111,112,113,114,115,116,117].
Hsp90	- cytoplasm; - ER; - mitochondria; - extracellular environment [94,118].	- Aβ [43].	- folding of many proteins [32]; - refolding of denatured proteins after stress [32]; - signal transduction [118]; - activation of immune system [97]; - activation of microglial phagocytosis [119].	- AD [43].	- interaction with the nucleotide-binding pocket [19]; - binding of Hsp90 C-terminal domain [19,133,134]; - modulation of Hsp90 functions through co-chaperones [137,138].
